# A Patient With Thiamine Deficiency Exhibiting Muscle Edema Suggested by MRI

**DOI:** 10.3389/fneur.2018.01083

**Published:** 2018-12-11

**Authors:** Kenichiro Murate, Yasuaki Mizutani, Toshiki Maeda, Ryunosuke Nagao, Kouichi Kikuchi, Sayuri Shima, Yoshiki Niimi, Akihiro Ueda, Shinji Ito, Tatsuro Mutoh

**Affiliations:** Department of Neurology, Fujita Health University School of Medicine, Toyoake, Japan

**Keywords:** thiamine deficiency, neuropathy, myalgia, magnetic resonance imaging, muscle edema

## Abstract

Myalgia is sometimes observed in patients with thiamine-deficiency neuropathy. However, the detailed mechanism(s) underlying muscular manifestations have been poorly elucidated. We herein report a possible patient with thiamine-deficiency neuropathy exhibiting muscle weakness and myalgia in lower limbs. The patient exhibited abnormal muscle signal intensities on MRI corresponding to the site of myalgia. After thiamine replacement therapy, rapid improvement of clinical symptoms and abnormal MRI findings were observed. Muscle MRI findings in this case implicated the possible mechanism of myalgia observed in patients with thiamine deficiency neuropathy.

## Background

Thiamine deficiency is known to cause various disorders including heart failure, peripheral neuropathy, and central nervous system disorder (1, 2). Thiamine-deficient patients often complain myalgia but the detailed mechanism(s) underlying muscular manifestations has been poorly elucidated (1, 3). We herein report a case of thiamine deficiency neuropathy exhibiting interesting findings on muscle magnetic resonance imaging (MRI).

## Case Report

A 34-year-old woman was admitted to our hospital with no relevant past medical history. She first noticed lumbago and pain in her legs 1 month prior to admission. 2 weeks later, she became aware of muscle weakness of the lower limbs. She had been eating a balanced diet and drinking moderately. She had no family history of similar symptoms. Her general physical findings were unremarkable, with no signs of heart failure. Neurological examination disclosed no disturbance of consciousness or cranial nerve abnormalities. No muscle weakness was present in her neck or upper extremities, but mild weakness was evident in the lower limbs. During daily physical activities, she experienced severe pain in the gastrocnemius muscles with tenderness. Deep tendon reflexes in the lower legs were hypoactive without pathological reflexes. Mild distal-dominant hypoesthesia in bilateral legs was also noted.

Blood examination revealed mild hepatic dysfunction. Serum creatine kinase (CK) was not elevated at 39 U/ L (normal, 45–163). Neither myoglobin nor aldolase was elevated at 13.1 ng/ml (normal, < 106.0) and 5.1 U/L (normal, 2.1–6.1), respectively. The serum thiamine level was 12 ng/ml (normal, 24–66). Regarding the low levels of thiamine, we examined its level three times and confirmed to be low in all the three determinations. We conducted investigations including gastrointestinal endoscopy and blood examination to explore the cause of thiamine deficiency such as malabsorption, obstruction, hyperthyroidism, and adrenal insufficiency. However, we could not identify the obvious cause for the low levels of thiamine. Levels of vitamin B2, B12, and folic acid were within normal range. All the autoantibodies tested in the present were negative, including antinuclear antibodies, anti-neutrophil cytoplasmic antibodies, paraneoplastic autoantibodies (Hu, Yo, Ri, Ma1, Ma2, and CV-2, amphiphysin), and anti-cardiolipin antibodies. The cerebrospinal fluid was normal, and the IgG index was 0.59.

On admission, needle electromyography of tibialis anterior and gastrocnemius exhibited a decreased recruitment pattern with mostly normal motor unit potentials in voluntary contraction, although these muscles showed the fibrillation potentials and positive sharp waves as spontaneous activities, suggesting active denervation. Moreover, early recruitment was not observed. The findings of chronic denervation were not observed. These findings in needle electromyography were compatible with acute motor axonopathy. In nerve conduction studies, motor conduction velocities and the compound muscle action potential amplitude (CMAP) were within normal range in the extremities, but mild generalized large fiber sensory axonopathy was revealed. To summarize these findings, neurophysiological investigations indicated sensorimotor axonopathy with active denervation of motor nerves in lower limbs.

**Figure 1:**
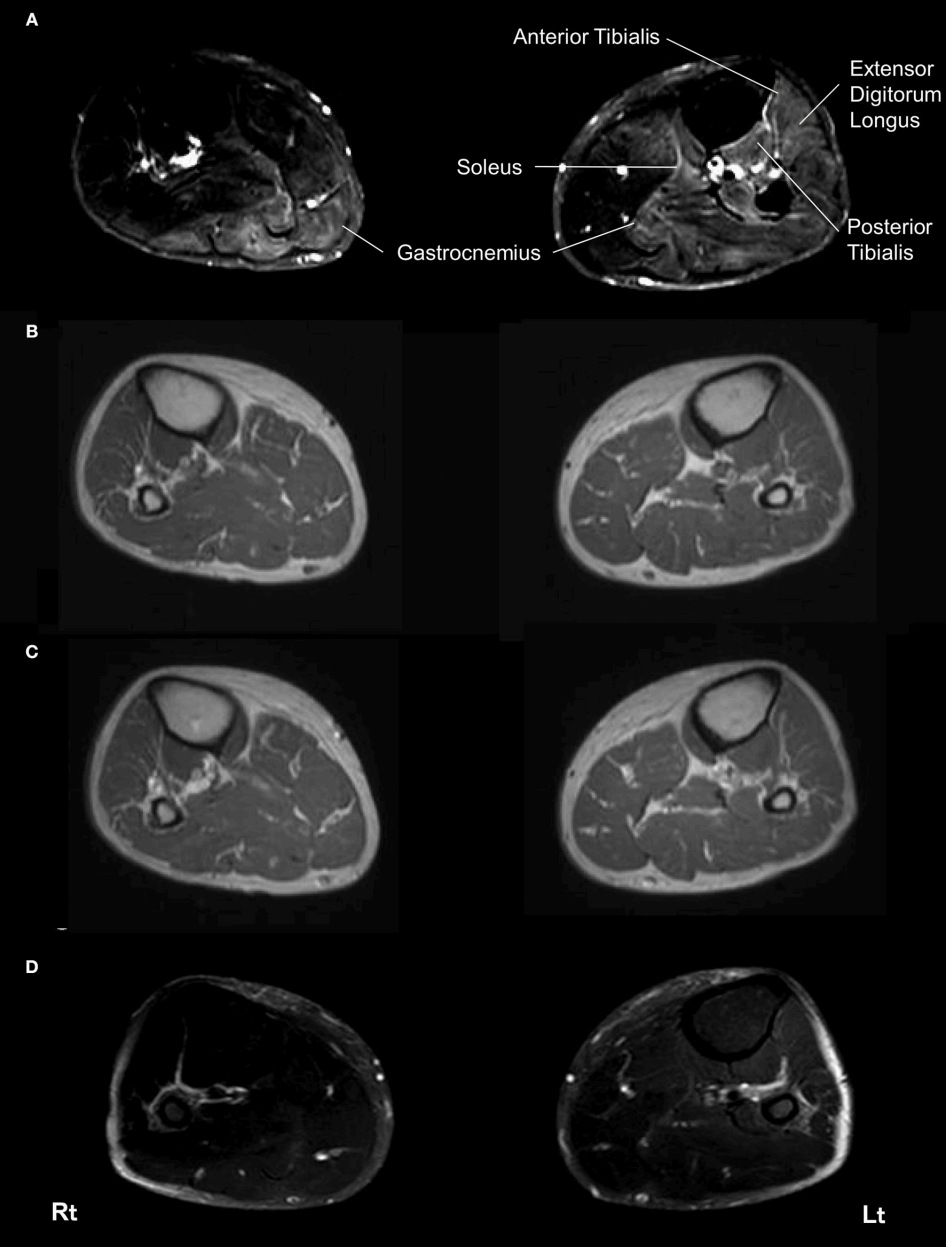
Magnetic resonance imaging of the bilateral lower limbs muscles. On admission, spectral attenuated inversion recovery (SPAIR) T2-weighted images of muscle MRI revealed left-side dominant high intensities throughout the bilateral gastrocnemius muscles, left soleus muscle, left anterior and posterior tibialis muscles, and left extensor digitorum longus muscle at this level **(A)**. No abnormal lesions of these muscles were seen in a T1-weighted image on admission **(B)**. No gadolinium contrast enhancement was observed in these muscles on admission **(C)**. SPAIR T2-weighted images showed improvement of the abnormal intensities in these muscles 4 months later **(D)**. Rt, right side; Lt, left side.

Muscle MRI on admission revealed patchy high signal intensities of various degree in her bilateral gastrocnemius muscles, left soleus muscle, left anterior and posterior tibialis muscles, and left extensor digitorum longus muscle on spectral attenuated inversion recovery (SPAIR) T2-weighted images (Figure 1A), whereas there were no abnormal intensities on T1-weighted images and no gadolinium contrast enhancement (Figures 1B,C). Brain and spinal MRI findings were normal.

Under the tentative diagnosis of thiamine deficiency neuropathy, massive intravenous thiamine replacement therapy was initiated at a dose of 300 mg/day. Myalgia and weakness in the lower limbs and gait disturbance markedly improved 2 days after therapy was initiated. MRI findings on SPAIR T2-weighted images improved within 1 month (Figure 1D). We have measured the serum thiamine level several times after her recovery and confirmed that the serum thiamine levels were normalized. Abnormal needle electromyography findings were completely normalized within 2 months. The patient was discharged from the hospital on day 17 and is currently followed up to 13 months in the outpatient clinic without relapse.

## Discussion

In the present case, although her levels of myogenic enzymes were not elevated, she had severe muscle pain and motor weakness. Neurological, electrophysiological, MRI findings and excellent responses to thiamine supplementation suggested a diagnosis of thiamine deficiency neuropathy.

According to the previous reports, thiamine deficiency neuropathy exhibits motor-dominant axonopathy (4). In the present case, the findings of active denervation were observed in the needle electromyography, but CMAP were within normal range in the nerve conduction studies. Concerning about the electrophysiological abnormalities in the subclinical phase of thiamine deficiency neuropathy, the findings of active denervation potential in needle electromyography were demonstrated to show higher sensitivities than the finding of reduced CMAP (5). Thus, the electrophysiological pattern of this case was considered to be compatible with the abnormalities in the very early phase of thiamine deficiency neuropathy.

Till date, thiamine deficiency is considered to be rare in the western world. However, it was reported that the frequency of thiamine deficiency was higher than conventionally thought due to excessive intake of soft drinks, junk food, and high-carbohydrate alcoholic beverages, such as beer, and long-term administration of furosemide (6, 7). Thiamine deficiency neuropathy was reported to exhibit acutely progressive pattern. Furthermore, it typically exhibits symptoms such as weakness, paresthesia, and myalgia, and presents a pattern of motor nerve-dominant involvements (4). Some previous cases showed rapidly progressive motor weakness mimicking Guillain-Barré syndrome (8). Other metabolic neuropathies including alcoholic neuropathy were reported to exhibit sensory-dominant and slowly progressive pattern as a main clinical phenotype, and do not fit those of our case (4, 9).

Of particular note, muscle MRI initially showed high-signal-intensity lesions across her several muscles in the lower limbs, especially with fat suppression imaging, although T1-weighted images were unremarkable. Previous studies have indicated that high signal intensities on T1-weighted images of muscle reflect fatty infiltration or subacute hematoma, while high intensities on T2-weighted images are assumed to indicate edema, respectively (10–12). Furthermore, acute denervation results in an increased signal intensity in skeletal muscles on fat-suppressed T2-weighted images (13–15). Particularly, in this case, muscle MRI findings of the lower legs exhibited patchy high signal intensities of variable degree on fat-suppressed T2-weighted images in common with abnormal MRI findings in the acute phase of severe Guillain-Barrē syndrome (16). The previous study has suggested the possibility that muscle edema could be derived from the enlargement of the intramuscular capillary bed in acutely denervated muscles, whereas in chronic phase of denervation, muscle atrophy and replacement with fat were reported to dominate the imaging findings (15). Under these considerations, we speculate that myalgia in this case might arise from muscle edema due to acute denervation due to thiamine deficiency.

Thus, we detected for the first time muscle MRI abnormalities on the sites corresponding to the location of myalgia. Findings of muscle MRI in this patient suggested local edema in muscles resulting in myalgia, which might be due to active denervation by rapid progressing thiamine deficiency neuropathy. Further investigation is needed to verify the effectiveness of muscle MRI in thiamine deficiency neuropathy.

In conclusion, the present case suggests that thiamine deficiency neuropathy, especially in the early course of the disease can exhibit abnormal muscle MRI findings indicating local edema without any elevation of serum myogenic enzymes. Therefore, muscle MRI examination can be taken into consideration for the diagnosis in thiamine deficiency neuropathy patients with myalgia and immediate treatment with thiamine replacement therapy are necessary for better outcomes.

## Ethics Statement

The case report has been performed in accordance with the ethical standards laid down in the 1964 Declaration of Helsinki and its later amendments. Consent was obtained for publication of the case details.

## Author Contributions

All the authors took care of patient management and made decisions about patient treatment. KM and YM conceived the idea, revised all the literature, and wrote the manuscript. ToM and SI collected the clinical data. RN analyzed and interpreted muscle imaging. KK, SS, YN, and AU collected the clinical data and contributed to the writing of the manuscript. TaM contributed to the intellectual contents, drafting the manuscript, and revision of the manuscript.

### Conflict of Interest Statement

The authors declare that the research was conducted in the absence of any commercial or financial relationships that could be construed as a potential conflict of interest.

## References

[1] Ohnishi A Tsuji S Igisu H Murai Y Goto I Kuroiwa Y. Beriberi neuropathy. Morphometric study of sural nerve. J Neurol Sci. (1980) 45:177–90. 10.1016/0022-510X(80)90164-1 7365498

[2] Harper CG Giles M Finlay-Jones R. Clinical signs in the Wernicke-Korsakoff complex: a retrospective analysis of 131 cases diagnosed at necropsy. J Neurol Neurosurg Psychiatry (1986) 49:341–5. 10.1136/jnnp.49.4.341 3701343 PMC1028756

[3] Koike H Misu K Hattori N Ito S Ichimura M Ito H. Postgastrectomy polyneuropathy with thiamine deficiency. J Neurol Neurosurg Psychiatry (2001) 71:357–62. 10.1136/jnnp.71.3.357 11511711 PMC1737557

[4] Koike H Iijima M Sugiura M Mori K Hattori N Ito H. Alcoholic neuropathy is clinicopathologically distinct from thiamine-deficiency neuropathy. Ann Neurol. (2003) 54:19–29. 10.1002/ana.10550 12838517

[5] Djoenaddi W Notermans SLH Lilisantoso AH. Electrophysiologic examination of sunclinical beriberi polyneuropathy. Electromyogr. Clin. Neurophysiol. (1995) 35:439–42. 8549435

[6] Kawai C Wakabayashi A Matsumura T Yui Y. Reappearance of beriberi heart disease in Japan : a study of 23 cases. Am J Med. (1980) 69:383–6. 10.1016/0002-9343(80)90008-X 7416185

[7] Seligmann H Halkin H. Thiamine deficiency in patients with congestive heart failure receiving long-term furosemide therapy: a pilot study. Am J Med. (1991) 91:151–5. 10.1016/0002-9343(91)90007-K 1867241

[8] Murphy C Bangash IH Varma A. Dry beriberi mimicking the Guillain-Barre syndrome. Pract Neurol. (2009) 9:221–4. 10.1136/jnnp.2009.182097 19608771

[9] Koike H Takahashi M Ohyama K Hashimoto R Kawagashira Y Iijima M. Clinicopathologic features of folate-deficiency neuropathy. Neurology (2015) 84:1026–33. 10.1212/WNL.0000000000001343 25663227

[10] Hollingsworth KG de Sousa PL Straub V Carlier PG. Towards harmonization of protocols for MRI outcome measures in skeletal muscle studies: consensus recommendations from two TREAT-NMD NMR workshops, 2 May 2010, Stockholm, Sweden, 1-2 October 2009, Paris, France. Neuromuscul Disord. (2012) 22(Suppl. 2):S54–67. 10.1016/j.nmd.2012.06.005 22980769

[11] Degardin A Morillon D Lacour A Cotten A Vermersch P Stojkovic T. Morphologic imaging in muscular dystrophies and inflammatory myopathies. Skeletal Radiol. (2010) 39:1219–27. 10.1007/s00256-010-0930-4 20449587

[12] Tasca G Monforte M Iannaccone E Laschena F Ottaviani P Leoncini E. Upper girdle imaging in facioscapulohumeral muscular dystrophy. PLoS ONE (2014) 9:e100292. 10.1371/journal.pone.0100292 24932477 PMC4059711

[13] Lisle DA Johnstone SA. Usefulness of muscle denervation as an MRI sign of peripheral nerve pathology. Australas Radiol. (2007) 51:516–26. 10.1111/j.1440-1673.2007.01888.x 17958685

[14] Kamath S Venkatanarasimha N Walsh MA Hughes PM. MRI appearance of muscle denervation. Skeletal Radiol. (2008) 37:397–404. 10.1007/s00256-007-0409-0 18360752

[15] McMahon CJ Wu JS Eisenberg RL. Muscle edema. AJR Am J Roentgenol. (2010) 194:W284–292. 10.2214/AJR.10.4247 20308472

[16] Sedano M Canga A de Pablos C Polo J Berciano J. Muscle MRI in severe Guillain-Barrē syndrome with motor nerve inexcitability. J Neurol. (2013) 260:1624–30. 10.1007/s00415-013-6845-y 23370612

